# Mobile Subcutaneous Calcinosis Cutis: A Case Report of a Mobile Solitary Subepidermal Calcified Nodule on a Woman’s Leg and a Review of Mobile Subcutaneous Tumors

**DOI:** 10.7759/cureus.37623

**Published:** 2023-04-15

**Authors:** Olive C Osuoji, Nathan S Uebelhoer, Christof P Erickson, Antoanella Calame, Philip R Cohen

**Affiliations:** 1 Dermatology Clinical Research, University of California San Diego, San Diego, USA; 2 Dermatologic Surgery, San Diego Family Dermatology, National City, USA; 3 Dermatology, Compass Dermatopathology, San Diego, USA; 4 Dermatology/Dermatopathology, Compass Dermatopathology, San Diego, USA; 5 Dermatology, Scripps Memorial Hospital, La Jolla, USA; 6 Dermatology, University of California Davis Medical Center, Sacramento, USA

**Keywords:** subepidermal, subcutaneous, nodular, mobile, disease, cutis, cutaneous, calcinosis cutis, calcinosis, calcified

## Abstract

Calcinosis cutis describes the deposition of calcium in the dermis. A case of a 69-year-old woman with idiopathic calcinosis cutis that presented as a mobile subcutaneous nodule is described. The patient had an asymptomatic, firm, mobile subcutaneous nodule on her right lower leg of at least six months duration. The nodule could be easily moved from one location to another. An incisional biopsy was performed. Microscopic examination of the tissue specimen showed islands of basophilic calcium material in dense sclerotic dermal connective tissue establishing the diagnosis of calcinosis cutis. Mobile solitary calcification is an unusual presentation of idiopathic calcinosis cutis. In addition to idiopathic calcinosis cutis, benign mobile subcutaneous tumors have also been derived from adnexal structures of hair follicles and adipose tissue. Hence, not only idiopathic calcinosis cutis, but also subepidermal calcinosis in the ocular adnexa, proliferating trichilemmal cyst with focal calcification, and mobile encapsulated adipose tissue can present as a mobile subcutaneous nodule. The features of idiopathic calcinosis presenting as a mobile subcutaneous nodule as well as the characteristics of other benign mobile subcutaneous tumors are reviewed.

## Introduction

The deposition of calcium in the skin is referred to as calcinosis cutis. Five types of calcinosis cutis have been observed. These include calciphylaxis, dystrophic calcinosis, iatrogenic calcinosis, idiopathic calcinosis, and metastatic calcinosis [1-4].

Mobile subcutaneous tumors are uncommon. They can be derived from adipose tissue, adnexal structures, or calcium. These benign neoplasms can be manipulated to travel from one area to another [5-14].

A woman presented with an asymptomatic mobile subcutaneous nodule; an incisional biopsy revealed a subepidermal calcific nodule. The history of this idiopathic variant of calcinosis cutis is summarized. Also, the features of other mobile subcutaneous nodules are reviewed.

## Case presentation

A 69-year-old Caucasian woman presented for evaluation of a lesion beneath the skin on her right leg. The patient noticed the new mass after experiencing a fall six months earlier. However, she commented that the lesion could have been present prior to her fall and that she had not been aware of it until after the fall.

She had a 50-pack-year history of smoking cigarettes and also had multiple medical comorbidities. These included asthma, chronic pain, depression, gastroesophageal reflux disease, hyperlipidemia, macular degeneration, osteoporosis, and prediabetes; in addition, she recently had a keratoacanthoma removed from her right leg. There was no history of immunosuppression or collagen vascular disease. Her medications included an albuterol inhaler, alendronate, aspirin, flonase, omeprazole, trazodone, tylenol, and simvastatin. Her serum chemistries, including calcium and phosphate, were normal.

**Figure 1:**
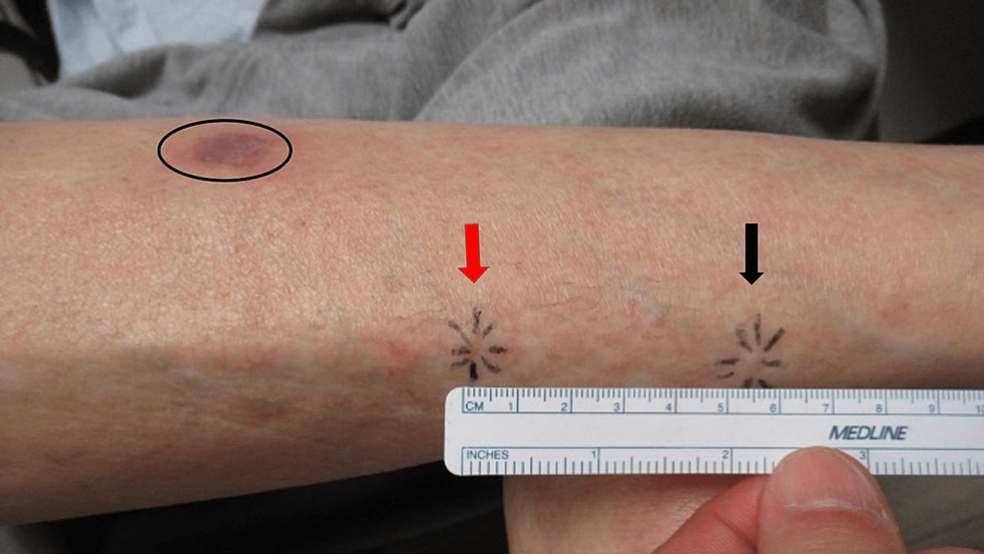
Clinical presentation of a mobile subepidermal calcified nodule. The pretibial right leg of a 64-year-old woman shows the original proximal location of the subcutaneous nodule (red arrow pointing to site demarcated by thin lines of black ink) and the distal location 5.5 centimeters away to which it has moved (thick black arrow pointing to subepidermal nodule demarcated by thin lines of black ink). The site of her prior keratoacanthoma appears as a red-purple-colored patch (within the black oval).

A complete cutaneous examination, from head to toe, was performed. An 18 x 18 millimeter red scar was present on her right leg at the excisional biopsy site of the keratoacanthoma (Figure 1). The tissue specimen had clear margins; there was no lymphadenopathy in the inguinal, axillary, or neck regions. Additional treatment of the tumor site (such as an excision with wider margins of normal skin) was discussed. She elected to have the site observed clinically without any additional intervention.

In addition, a new 3 x 1 millimeter subcutaneous mobile nodule was discovered (Figure 1). The nodule could be moved several centimeters distally and also return to its original site of origin when it was gently pushed (Video 1). Indeed, the nodule was able to be moved at a total distance of 5.5 centimeters.

**Video 1:** Mobile calcinosis cutis The subepidermal calcified nodule can easily be moved from a proximal to a distal location on the woman's right pretibial leg.

**Figure 2:**
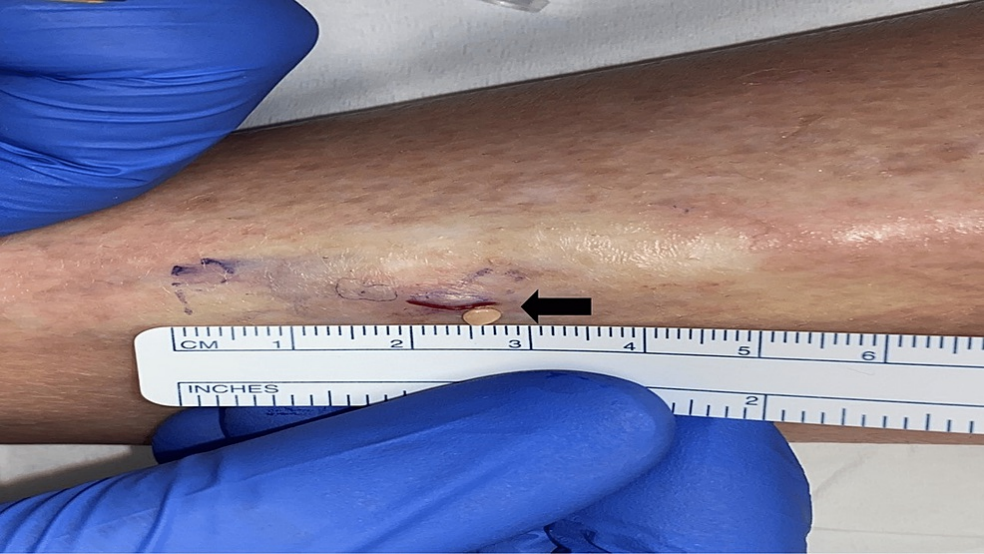
Incisional biopsy of proximal right pretibial leg A 7 millimeter incision was performed on the proximal pretibial right leg of the woman with the mobile subcutaneous nodule. A 3 x 1 millimeter yellow firm nodule was expressed from the proximal edge of the incision (black arrow).

An incisional biopsy was performed. A yellow nodule was easily expressed from the biopsy site (Figure 2). Interrupted sutures were used to close the wound.

**Figure 3:**
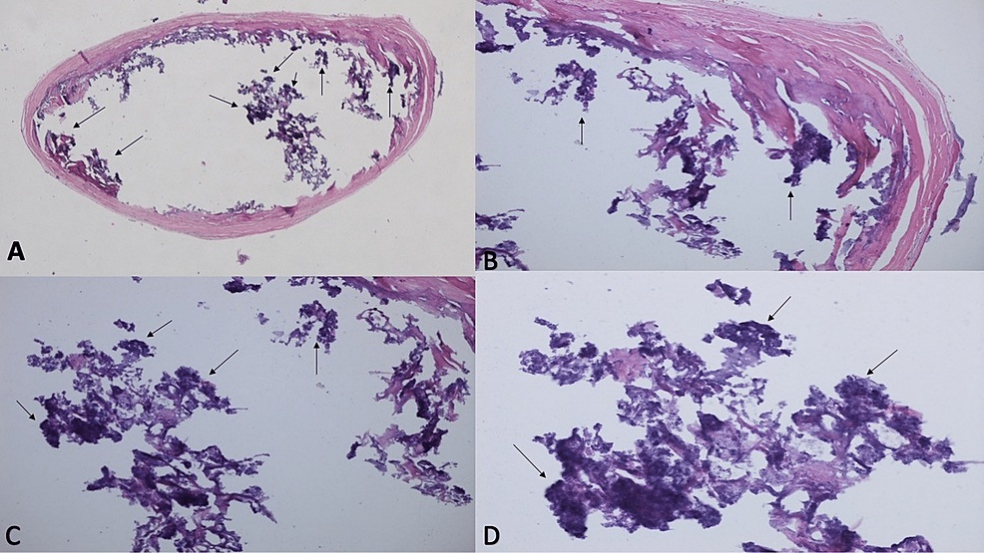
Microscopic presentation of a mobile subepidermal calcified nodule Distant (A) and closer (B, C, and D) views of the tissue specimen show a circumscribed nodule surrounded by dense sclerotic connective tissue with islands of basophilic calcium (black arrows) (hematoxylin and eosin: A, x 2; B, x 10; C, x 20; D, x 40).

A microscopic examination of the tissue specimen was performed. Hematoxylin-and-eosin-stained sections showed a dermal nodule that was surrounded by sclerotic collagen. Within the nodule were multiple islands of calcium presenting as basophilic material (Figure 3).

Correlation of the history, clinical presentation, and pathology established the diagnosis of idiopathic calcinosis cutis presenting as a mobile solitary subepidermal calcific nodule.

## Discussion

Calcinosis cutis -- a group of diseases characterized by calcium deposits in the dermis of the skin -- can present as either calciphylaxis, dystrophic calcification, iatrogenic calcification, idiopathic calcification, or metastatic calcification. Metastatic calcification is the deposit of calcium in normal cutaneous or subcutaneous tissue. It occurs in the setting of elevated levels of serum calcium and/or phosphate [1-4].

Dystrophic calcification is the deposition of calcified material in injured tissue; it is associated with normal serum calcium and phosphate levels. Dystrophic calcification is the most common presentation of calcinosis cutis. It can occur in association with autoimmune connective tissue diseases such as dermatomyositis, lupus erythematosus, and systemic sclerosis [3,4,15].

Calciphylaxis results in a cutaneous ischemic infarct that is caused by the occlusion of blood vessels in the subcutaneous fat and dermis by calcification. It is seen primarily in uremic renal failure patients; however, non-uremic calciphylaxis has also been reported. Sodium thiosulfate has been successfully used to treat some of these individuals [1,4].

Iatrogenic calcification occurs following diagnostic or therapeutic uses of substances containing calcium or phosphate. There are reported cases of calcinosis cutis complicating intravenous calcium chloride or calcium gluconate therapy leading to tissue damage at the site of extravasated calcium. Iatrogenic calcification can be prevented by diluting the calcium solution and lowering phosphorus levels before administration [1,4]. 

Idiopathic calcinosis cutis is characterized by normal serum calcium and phosphorus levels. It includes nodular calcinosis of Winer (which is also known as calcified subepidermal nodule), scrotal calcinosis, and tumoral calcinosis [1,2]. Our patient had the calcified subepidermal nodule variant of idiopathic calcinosis cutis.

The calcified subepidermal nodule was initially recognized by Winer in 1952 as a solitary congenital nodular calcification of the skin [16]. He reported the condition in three children who ranged in age from 10 to 20 months. However, reports by other investigators demonstrated that the lesions were not always solitary and were frequently not congenital (Table 1) [2,13,16,17].

**Table 1:** History of solitary nodular calcification CR, current report; Ref, references.

Author	Year	Comment	Ref
Winer	1952	Three patients (two girls and one boy) described with localized nodular congenital calcinosis of the skin. The children ranged in age from 10 to 20 months. All of the lesions were successfully excised.	[16]
Woods and Kellaway	1963	A review of 20 new patients with cutaneous calculi presenting as subepidermal calcified nodules. The age at which the calculus was first noticed ranged from birth to 56 years. No recurrences were noted after the lesions were removed.	[17]
Azon-Masoliver et al.	1989	Two patients with solitary congenital nodular calcification of Winer were reported. Their lesions were located on the ear.	[2]
Khine et al.	2018	A retrospective systemic review of 53 patients with mobile subepidermal calcinosis located in the ocular adnexa.	[13]
Osuoji et al.	2022	A report of a 69-year-old woman with mobile calcinosis cutis presenting as a subcutaneous nodule. Her lesion was successfully removed without recurrence.	CR

In 1963, Woods and Kellaway introduced the nomenclature of subepidermal calcified nodules [17]. Indeed, in addition to solitary congenital nodular calcification and subepidermal calcified nodules, this benign calcium deposition has been described as cutaneous calculi, idiopathic calcinosis cutis, localized cutaneous calcinosis, and solitary calcification. Subepidermal calcified nodule most commonly affects otherwise healthy children and adolescents with a mean age of 8.4 years. The male-to-female ratio is 2:1 [2,13,16,17].

Subepidermal calcified nodule typically presents as a single, small, asymptomatic, painless, hard, freely mobile yellow-white papule on the head and neck region with the eyelid being the most common site. Rare locations such as fingers, knees, oral mucosa, palms, penis, soles, and toes have been documented. The appearance of the overlying epidermis can be verrucous, hyperkeratinized, or papillomatous, and can vary in color from yellow, white, gray, or pink [3,17].

There is no history of trauma, systemic disease, or abnormal laboratory findings in patients with the subepidermal calcified nodule. Notably, calcium, phosphate, and parathyroid hormone levels are typically in the normal range. When it presents as a single nodule, it can clinically mimic a calcifying pilomatrixoma, epidermal inclusion cyst, hemangioma, seborrheic keratosis, or trichilemmal cyst [12]. When it presents as multiple nodules, some of the conditions in the differential diagnosis include milia, molluscum contagiosum, verrucous papilloma, and xanthoma [13,17].

The woman in this report had an acquired asymptomatic subcutaneous mobile nodule. It could readily be moved from a proximal site on her leg to a more distal location and then it could be moved back to the original site of origin. An incisional biopsy not only removed the benign lesion but also established the diagnosis of a mobile subepidermal calcific nodule.

In contrast to our patient’s lesion occurring on her leg, a retrospective study of 53 patients observed mobile subepidermal calcinosis to be localized to the ocular adnexa. Men were affected more commonly than women (in a 3:1 ratio) and most (89%) of the patients are 21 years of age or younger. In addition, the majority of patients (63%) were non-Caucasian [13].

The lesions of mobile subepidermal calcinosis localized to the ocular adnexa appeared as a solitary, hard, white, or yellow nodules with papillomatous features and were frequently located on the upper eyelid. Most of the patients (82%) had a single lesion [13]. Recently, a solitary lesion of subepidermal calcinosis has been described in the lower eyelid of a 13-year-old boy with neurofibromatosis type 1 [14].

Several investigators have proposed various mechanisms of pathogenesis for subepidermal calcified nodules. Woods and Kellaway suggested two mechanisms for the formation of the subepidermal calcified nodule. The first mechanism is that calcified granules within the stroma create a larger calcified mass through calcification and degeneration of the stroma. Alternatively, the large calcified lesion occurs first and its subsequent reabsorption leaves the calcified granules that form the calcified nodule with associated tissue changes [17].

Other researchers have proposed calcification of pre-existing skin structures. It was thought that cutaneous calculi might be derived from milia. It has also been postulated that they are hamartomas of sweat duct origin, most likely syringomas, with calcification. Alternatively, they may result from calcium deposits in a preexisting nevus [2].

In 1980, Tezuka explained the mechanism that forms a subepidermal calcified nodule to be secondary calcinosis resulting from the deposition of calcium and phosphate after the degranulation of mast cells [17,18]. Calcification of subcutaneous fat necrosis and trauma are other proposed hypotheses. Indeed, to date, the definitive etiology and pathogenesis of subepidermal calcified nodules are still unknown [2].

For a patient with calcinosis cutis, a series of laboratory tests and imaging studies are recommended to identify the type of calcification and determine its etiology. In all patients, serum calcium, inorganic phosphate, alkaline phosphatase, and albumin levels should be measured; serum calcium or phosphate or both are elevated in metastatic calcification. All of these laboratory tests were normal in our patient [1,4].

Definitive diagnosis of calcinosis cutis is by biopsy. Microscopic examination shows calcium deposition in the dermis, with or without chronic lymphocytic inflammation. Often a histiocytic reaction to the dermal deposits is also present. Indeed, chronic inflammation and foreign body giant cell reactions are prominent in children and adolescents; in contrast, these pathologic changes may be mild or absent in adults [1,14].

Surgical excision is the first-line therapy for idiopathic calcinosis cutis, as was done in our patient. Indeed, an excisional biopsy -- for diagnostic purposes and treatment -- is an excellent approach when the clinical diagnosis of calcinosis cutis is suspected. Recurrence can occur after incomplete excision [19].

Indications for the treatment of subepidermal calcified nodules are impairment of function due to location and the presence of pain. However, cosmetic concerns are the most common reasons for treatment. In addition to surgical removal, intralesional corticosteroid injection is another treatment modality for subepidermal calcified nodules [19].

The differential diagnosis of benign mobile subcutaneous tumors is summarized in Table 2 [5-14]. It not only includes neoplasms derived from calcification but also tumors originating from adnexal structures such as hair follicles and adipose tissue. Indeed, in addition to a subepidermal calcific nodule, the neoplasms include mobile encapsulated adipose tissue (MEAT), proliferating trichilemmal cyst with focal calcification, ossifying plexiform tumor, and subepidermal calcinosis in the ocular adnexa.

**Table 2:** Differential diagnosis of mobile subcutaneous tumors CR, current report; MEAT, mobile encapsulated adipose tissue; Ref, references. ^a^This tumor has also been referred to as abacus tumor, encapsulated fat necrosis, mobile encapsulated adipose tissue, mobile encapsulated lipoma, and nodular cystic fat necrosis. ^b^This tumor has also been referred to as cutaneous calculi, localized calcinosis cutis, and mobile calcinosis cutis.

Tumor	Comment	Ref
Mobile encapsulated adipose tissue^a^	The tumor is referred to by the acronym MEAT. It consists of adipose tissue. Frequently, patients with MEAT present with multiple tumors.	[5-8]
Ossifying plexiform tumor	A benign tumor that has been observed on the fingers of women. Microscopic examination shows a myxomatous stroma containing not only plexiform nests of spindle to epithelioid cells but also foci of ossification.	[9-11]
Proliferating trichilemmal cyst with focal calcification	A 64-year-old man presented with a large (4 x 5 x 6 centimeter) tumor that was mobile over the subcutaneous tissue of his posterior scalp of approximately 30 years duration. Excisional biopsy showed a ruptured proliferating trichilemmal cyst with focal calcification.	[12]
Subepidermal calcific nodule^b^	A solitary asymptomatic papule usually located on the head and neck region. However, they have been observed at other sites.	CR
Subepidermal calcinosis in the ocular adnexa	These are similar in morphology and pathology to a subepidermal calcific nodule. However, they most commonly localized to the upper eyelid and are multiple in nearly 20% of affected patients.	[13,14]

Patients with MEAT can present with multiple lesions. MEAT is also referred to by several designations. The adipose tumors of MEAT were initially reported as “abacus tumors,” a name coined to describe a marble-like, subcutaneous mass that is freely mobile, often over distances of several centimeters. Most of the lesions occur in regions vulnerable to trauma, such as the lower and upper extremities. They have also been occasionally described in the peritoneum [5-7].

Subsequently, these adipose tumors have been referred to as mobile encapsulated lipoma, nodular cystic fat necrosis, encapsulated fat necrosis, and encapsulated adiponecrosis [5,6]. They are usually observed in healthy adolescent boys or middle-aged women [7,8]. In contrast to the solitary lesions usually observed in individuals with subepidermal calcific nodules and often in patients with subepidermal calcinosis in the ocular adnexa, patients with MEAT can present with multiple lesions. For example, a 22-year-old man with nodular cystic fat necrosis presented with two mobile, rice-sized, deep-seated papules on his right shin for 10 years after trauma [7].

More recently, MEAT was proposed as a more accurate description [5]. The tumors consist of solitary or multiple, 2 to 35 millimeter, subcutaneous, often mobile, papulonodules, usually on the lower extremities, but occasionally on the arms, forearms, and trunk. The lesions may be present for several weeks to years before excision, and trauma precipitates the onset of the lesions in approximately 30% of patients. The preferred treatment is surgical and a simple excision is usually sufficient [6-8].

Ossifying plexiform tumor is an extraordinarily rare benign cutaneous neoplasm. Although none of the lesions demonstrated reports of progression or metastasis, local recurrence of the tumor was documented in one patient. It presents as subcutaneous nodules on the fingers of women, measuring 1 to 2 centimeters in greatest dimension. The tumor has a distinctive histologic appearance typified by plexiform nests of cytologically bland spindle-to-epithelioid cells in a myxocollagenous stroma with associated foci of central ossification, which may range from osteoid to the mature bone [9-11].

A proliferating trichilemmal cyst is also known as either a pilar cyst or wen. They are slow-growing, rubbery, non-tender, mobile nodules typically located on the scalp. Microscopic examination can reveal focal calcification. Complete excision is recommended since the tumor has a potential for locally aggressive behavior and malignant transformation [12].

The woman in this report presented with an asymptomatic mobile nodule. The tumor was completely removed when an incisional biopsy was performed. Microscopic examination established the diagnosis of a benign subepidermal calcific nodule.

## Conclusions

Calcinosis cutis is a clinical entity characterized by the deposition of insoluble calcium salts in the skin and subcutaneous tissue. There are five variants of calcinosis cutis: calciphylaxis, dystrophic calcification, iatrogenic calcification, idiopathic calcification, and metastatic calcification. A woman presented with a subcutaneous and mobile nodule on her leg; an incisional biopsy not only removed the entire lesion but also established the diagnosis of the subepidermal calcific nodule type of idiopathic calcinosis cutis. In addition to a subepidermal calcific nodule, MEAT, proliferating trichilemmal cysts, ossifying plexiform tumor, and subepidermal calcinosis in the ocular adnexa can present as a mobile subcutaneous neoplasm. Surgical removal of the lesion and microscopic evaluation of the tissue specimen can differentiate the morphologically similar appearing lesions.
